# Urdu translation and cross-cultural validation of the stroke self-efficacy questionnaire

**DOI:** 10.1186/s12883-024-03704-1

**Published:** 2024-06-29

**Authors:** Waffa Uroose, Mehwish Ikram, Maryam Ikram, Syed Shaki ur Rehman, Marvi Asif, Hafiza Rabia Javed

**Affiliations:** Faculty of Rehabilitation and Allied Health Sciences, Riphah International University, Lahore, Pakistan

**Keywords:** Stroke, Self Efficacy, Translation, Validation, Cross-cultural, Psychometric properties

## Abstract

**Background:**

The Stroke Self-Efficacy Questionnaire (SSEQ) measures the self-confidence of the individual in functional activities after a stroke. The SSEQ is a self-report scale with 13 items that assess self-efficacy after a stroke in several functional domains.

**Objective:**

The purpose was to translate the Stroke Self-Efficacy Questionnaire into Urdu Language and to find out the validity and reliability of Urdu SSEQ among stroke patients.

**Methods:**

The cross-cultural validation study design was used. Following COSMIN guidelines, forward and backward translation protocols were adopted. After pilot testing on 10 stroke patients, the final Urdu version was drafted. A sample of 110 stroke patients was used to evaluate the validity and reliability of the SSEQ-U. Content and Concurrent validity were determined. The intraclass correlation coefficient and Cronbach’s alpha were used to measure internal consistency and test-retest reliability. Data analysis was performed using SPSS 25.

**Results:**

The final version was drafted after application on 10 stroke patients. Content validity was analyzed by a content validity index ranging from 0.87 to 1. The internal consistency was calculated by Cronbach’s alpha (α > 0.80). Test-retest reliability was determined by the Intra-class correlation coefficient (ICC_2,1_=0.956). Concurrent validity was determined by correlations with other scales by using the Spearman correlation coefficient; moderate to strong correlations (positive and negative) were found with the Functional Independence Measure (*r* = 0.76), Beck Depression Inventory (*r*=-0.54), Short Form of 12-item Scale (*r* = 0.68) and Fall Efficacy Scale (*r* = 0.82) with *p* < 0.05.

**Conclusion:**

The Urdu version was linguistically acceptable and accurate for stroke survivors for determining self-efficacy. It showed good content and concurrent validity, internal consistency and test-retest reliability.

## Introduction

Stroke is one of the common causes of death and disability [1]. Stroke incidence is expected to increase globally, particularly among the elderly. Nearly identical risk factors exist for stroke, coronary heart disease, and other vascular illnesses [2]. Activities of daily living (ADL) are negatively influenced by stroke in stroke victims, and independence in ADL has grown to be a significant concern in the immediate and ongoing post-stroke care [3]. Self-efficacy is the term used to describe one’s assurance and belief in one’s ability to carry out a task or action [4]. According to Bandura, verbal persuasion, direct mastery experience, vicarious experience, and physiological state are the four main sources of self-efficacy [5]. Jones also mentioned these guidelines and proposed that these sources may be used to support the tactics used in self-efficacy programs [6]. After leaving the hospital, stroke rehabilitation continues, emphasizing the need for patients to pick up new skills and learn how to use them to implement and sustain self-management [7]. The stroke rehabilitation programs facilitate stroke self-management, focused on increasing self-efficacy and preventing secondary stroke risk factors [7]. Self-management programmes are associated with quality of life, depression, activities of daily living, physical function and reduced risk of falls [6–8].

ADL functioning abilities are impaired in two-thirds of stroke survivors which affects their self-efficacy. These functional problems force them to depend on others for daily activities [8]. The degree of impairment varies from person to person and with the central nervous system regions that continuously sustain injury [9]. Physical limitations in walking and upper limb disabilities are the most prevalent impairments [10]. The impairments in ADLs among stroke survivors are thought to be caused by several factors, including delayed clinical presentation, a lack of advancement in therapeutic approach and depressive post-stroke symptoms such as a lack of inspiration, decreased cognitive ability and a sense of self-doubt [11]. A notable and significant stroke consequence that adversely impacts functional abilities is post-stroke depression (PSD) [11]. It is asserted that higher motivation and cognitive abilities play a major role in functional recovery after stroke [12]. The notion is further confirmed by the fact that improving motor skills after a stroke requires practice in most cognitive areas, including memory and judgment [13–16].

A 13-item Stroke Self-Efficacy Questionnaire created by Fiona Jones et al. in 2008 had good face validity and was useful for usage during the stroke recovery process [17]. Higher self-goals and a deeper commitment to achieving functional independence despite obstacles are indicators of greater self-efficacy [17–19]. Since its initial English development in 2008 [17], the SSEQ has been translated and cross-culturally validated in several languages, including Chinese, Italian, Portuguese, Turkish, Danish, Hausa and Arabic [20–27] but not in the Urdu language (the national language of Pakistan). Pakistan is a middle-income country with a population of > 20 million people. Global statistics show that the incidence of stroke patients is increasing constantly in the developing world [28], including Pakistan and neighbouring countries. Thus, it is very important to take this challenge seriously to overcome and address this alarming issue for the local population.

Patient-reported Outcome Measures (PROMs) or Self-reported Outcome Measures (SROMs) can be used to measure a patient’s subjective feelings [29]. This study aimed to translate and validate the Stroke Self-Efficacy Questionnaire (SSEQ) in Urdu Language. Self-Efficacy Questionnaire Urdu version will be helpful in self-judgment of one’s abilities in community-dwelling individuals of our country who sustained and survived a stroke. Another important need for translating this tool into Urdu is that SSEQ is in the English language, which could not be understood by Urdu-speaking people because their native language is Urdu.

## Methodology

### Study design/ sample/ sampling technique

It was a cross-cultural validation study. The data was collected from Divine Health & Fitness Centre, Lahore and Sheikh Zayed Hospital, Lahore, Pakistan. The non-probability convenience sampling technique was used for data collection.

### Inclusion and exclusion criteria of the participants

Patients diagnosed with stroke (late subacute to chronic stage), aged between 30 and 70 years (both genders) having undergone rehabilitation after stroke, without any cognitive issues (evaluated by the neurologist) and able to understand Urdu Language were included in the study.

Participants with stroke with some unstable medical conditions (diabetes, cardiovascular diseases and neurological conditions diagnosed by medical record) leading to fatigue were excluded. Participants with some language problems (aphasia) were also excluded.

### Stroke self-efficacy questionnaire (SSEQ)

The SSEQ is a self-report scale that assesses self-efficacy after a stroke in several functional domains. Individuals rate their level of confidence in their ability to accomplish each of the 13 items on a scale from 0 to 10, where 10 demonstrates a great degree of assurance. Rasch analysis of the original SSEQ was done by Riazi et al. who proposed a 4-point scale and zero shows as not confident and 3 as very confident. SSEQ has two separate dimensions of self-efficacy related to recovery and independence after stroke (activity and self-management). Activity dimension consists of 1 to 8 questions and others 9 to 13 related to self-management. SSEQ original has good validity and reliability > 0.90 and criterion validity with FES scale was > 0.80 [17–19].

### Translation methods

**Fig. 1:**
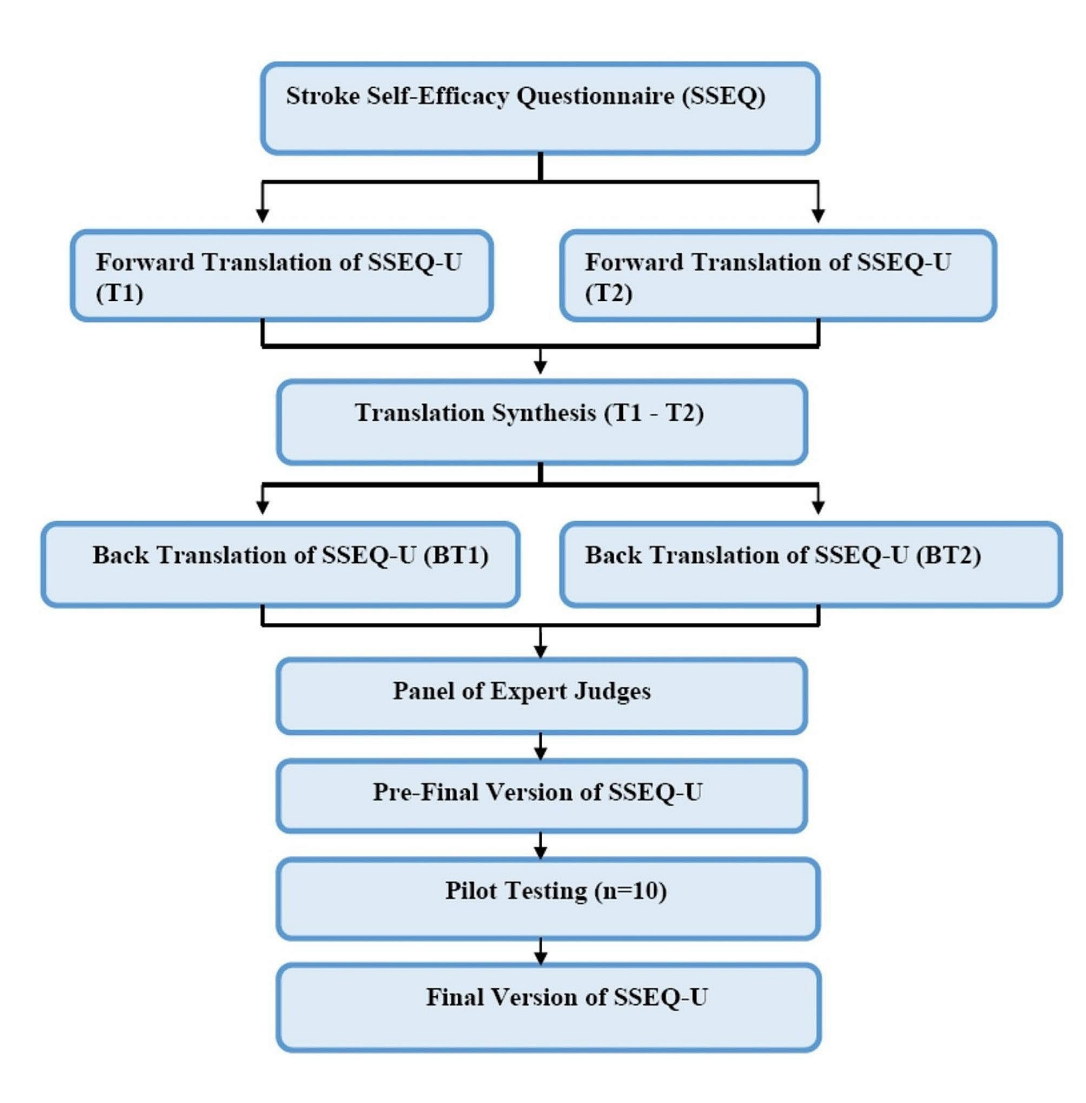
Flow chart of translation process

The Self-Efficacy Questionnaire was translated and cross-culturally validated using Beaton’s Guidelines and COSMIN guidelines [30–34]. Translation steps are shown in Fig. 1.

#### Forward Translation

The questionnaire was initially translated into Urdu from the original English version of SSEQ. The forward translations were done by two bilingual translators (English and Urdu). The study objective was completely explained to one of the translators who had a medical background as well. The other translator was not aware of the concept being quantified and neither had a medical background. The expert panel drafted the Urdu version of SSEQ.

#### Backward translation

Two bilingual translators (no expertise in medicine or knowledge of the ideas) back-translated the preliminary SSEQ-U into English. They did not have access to the original version of the SSEQ.

#### Reviewer Committee

A team of experts (two physical therapists (PhD, Professors) and one neurologist with experience > 10 years in neuro), comprising backwards and forward translators drafted the pre-final version of the SSEQ-U while emphasizing conceptual, semantic, and idiomatic similarity to the original SSEQ.

#### Pilot testing

Ten stroke survivors were in the pilot study (selected from Sheikh Zayed Hospital Lahore, Pakistan by following inclusion and exclusion criteria) and every single patient evaluated each element by using a scale of two values (understand and not understand) to express the understanding of queries. Satisfactory results of the pilot run were achieved, 7 participants rated the 13 items were comprehensive and 3 participants rated three questions were least understood.

#### Final version

The ultimate final version of SSEQ-U was drafted.

### Content validity index

The content validity index of the final SSEQ-U was determined by using the Waltz and Bausell method [35]. The content validity score was calculated by the Content Validity Index (CVI). Six physiotherapists (experience > 5 years, masters in neuromuscular physical therapy) were involved in this process. Relevant, clarity, simplicity and ambiguity, these four items were used for the content validity index (these four items were further assessed on the Likert scale) [35–37].

### Concurrent validity

The concurrent validity was determined by using gold-standard measures of basic daily living activities as well as the quality of life after stroke [24, 38, 39].

Concurrent validity was determined by correlating Urdu SSEQ with the Functional Independence Measure (FIM), Beck Depression Inventory (BDI), 12-Item Short Form Survey (SF-12) and Fall Efficacy Scale (FES). The readings on these scales were taken one time (at week 1 scoring).

FIM is used to measure the functional activities related to self-care, transfer and locomotion. Cognitive (memory, problem-solving) and communication skills (social interaction) can also be assessed. The total score varies from 18 to 126 and higher scores indicate more independencies [24, 40].

Beck Depression Inventory is a self-reported questionnaire consisting of 21 statement scores (0 to 63) and rated on 0 to 3 scales by the participants. A zero score indicates no depression while higher scores show greater severity of depression [24, 41].

The short-form survey of a 12-item scale is used to measure the quality of life. It consists of 12 items with two domains: physical and mental health. Higher scores indicate better quality of life [20, 42].

The Fall Efficacy Scale is used to determine the risk of falls during daily activities. It is a ten-item scale using a 10-point ordinal scale and the total score is 100. It can assess the risk of falls in different indoor and outdoor activities (such as bathing, reaching, sitting, walking, grooming etc.) [17, 43].

### Internal consistency and test-retest reliability

Internal consistency was determined for reliability analysis and test-retest reliability of SSEQ-U was determined by asking the patients to fill out the questionnaire with a gap of one week (week 1 and week 2 readings). Participants fill out the questionnaire under the supervision of the physiotherapist.

### Data analysis

Data was analyzed using SPSS version 25. The demographic data was analyzed by descriptive analysis (mean, standard deviation, percentages).

Mean, Standard Deviation (SD), Standard Error Mean (SEM), Minimal Detectable Change (MDC) and Cronbach’s Alpha of (SSEQ-U) week 1 and week 2 readings were determined (Formulas: SEM = S.D/√n, MDC = 1.96 × 2xSEM) at 95% confidence interval.

The Content Validity Index was used to determine content validity by using a Likert scale.

Concurrent validity was determined by comparing SSEQ-U with other scales (FIM, BDI, SF-12 and FES). Spearman correlation coefficient was used to determine the concurrent validity. Correlation values 0.40–0.60 are considered a moderate relationship; >0.80 is considered a strong relationship and > 0.90 is considered a very strong relationship [44].

Internal consistency and test-retest reliability were determined by Cronbach’s alpha (α) and intraclass correlation coefficient (ICC_2,1_) respectively. A value > 0.80 is considered good and > 0.90 is considered excellent [45, 46].

### Ethical concerns

The study was started after ethical approval with reference number REC/RCR & AHS/21/0246 from the Riphah International University, Lahore, Pakistan. Permission for the translation was taken from the author of SSEQ (original) [17].

## Results

### Demographic data

Demographic data of 110 stroke participants are shown in Table 1. The mean age of the participants was 48.65 ± 9.93. Both genders (57.3% males and 42.7% females) with ischemic (49%) and hemorrhagic (51%) strokes were involved in this study.

**Table 1:** Descriptive statistics of 110 patients with stroke

Descriptive statistics (110 Patients with stroke)	
Gender	n(%)
Male	63 (57.3%)
Female	47 (42.7%)
Age of Participants	Mean ± S.D (Years)
Male	48.6 ± 7.52
Female	49.9 ± 8.82
Affected side of body	n (%)
Right	59 (53.6%)
Left	51 (46.3%)
Type of stroke	n (%)
Ischemic	54(49.0%)
Hemorrhagic	56(50.9%)
Phases of Stroke	
Late sub-acute stage (3 to 6 months)	63 (57.2%)
Chronic stage (> 6 months)	47 (42.7%)

### Mean/ SEM/MDC and cronbach’s alpha

Mean, SD, SEM, MDC and Cronbach’s alpha of week 1 and week 2 readings (activity domain, self-management and total SSEQ-U scores) are shown in Table 2.

**Table 2:** Mean/ SEM/MDC and Cronbach’s Alpha of Week 1 and 2 readings of SSEQ-U

Week 1 (*n* = 110)	Activity domain	Self-Manangement	Total (SSEQ-U)
Mean ± SD	10.53 ± 3.02	6.26 ± 2.28	16.32 ± 3.45
SEM	0.28	0.21	0.39
MDC	1.09	0.82	1.52
Cronbach’s Alpha	0.85	0.86	0.88
Week 2 (*n* = 89)	Activity domain	Self-Manangement	Total (SSEQ-U)
Mean ± SD	10.26 ± 2.85	6.85 ± 2.37	16.85 ± 3.33
SEM	0.30	0.25	0.35
MDC	1.17	0.98	1.37
Cronbach’s Alpha	0.84	0.84	0.85

[Abbreviations: SD = Standard Deviation; SEM = Standard Error Mean; MDC = Mininmal Detectable Change]

SEM and MDC for the activity domain of SSEQ-U in week 1 (0.28, 1.09 points) and in week 2 (0.30, 1.17) respectively. SEM and MDC for the self-management domain of SSEQ-U in week 1 (0.21, 0.82 points) and in week 2 (0.25, 0.98) respectively. SEM and MDC for total SSEQ-U in week 1 (0.39, 1.52 points) and in week 2 (0.35, 1.37) respectively.

Internal consistency was determined by Cronbach’s alpha; week 1 (α = 0.885) and week 2 (α = 0.857).

### Content validity index (CVI)

The lower value was 0.87 and the highest value was 1 (maximum value). The maximum score was given to questions 1, 6, 7 and 10 by experts (Shown in Table 3).

**Table 3:** Internal consistency, test-retest reliability and content validity index

Test-Retest Reliability	Intraclass Correlation (ICC_2,1_)	95% Confidence Interval		Cronbach alpha (α)	CVI
		Lower Bound	Upper bound		
Question No 1	0.986	0.975	0.992	0.994	1.00
Question No 2	0.972	0.951	0.984	0.985	0.95
Question No 3	0.895	0.782	0.919	0.923	0.93
Question No 4	0.998	0.969	0.995	0.996	0.90
Question No 5	0.938	0.891	0.965	0.969	0.95
Question No 6	0.989	0.981	0.994	0.995	1.00
Question No 7	0.964	0.937	0.980	0.982	1.00
Question No 8	0.974	0.954	0.985	0.987	0.90
Question No 9	0.984	0.980	0.989	0.993	0.87
Question No 10	0.964	0.936	0.979	0.989	1.00
Question No 11	0.914	0.849	0.951	0.963	0.87
Question No 12	0.957	0.925	0.976	0.987	0.95
Question No 13	0.972	0.950	0.984	0.988	0.95
Total Score	0.956	0.934	0.976	0.985	0.90

[Abbreviations: ICC = Intraclass correlation coefficient; CVI = Content Validity Index]

### Concurrent validity

Concurrent validity was determined by correlating the FIM, BDI, SF-12 and FES with activity domain, self-management and total SSEQ-U. (Shown in Table 4)

**Table 4:** Correlations with other scales (Validity)

Concurrent Validity	Activity domain	Self-Management domain	Total SSEQ-U
SSEQ-U + FIM	*r* = 0.68 (*p* < 0.05)	*r* = 0.75(*p* < 0.05)	*r* = 0.76 (*p* < 0.05)
SSEQ-U + BDI	*r* = 0.34 (*p* = 0.234)	*r* = 0.27(*p* = 0.129)	*r*=-0.54 (*p* < 0.05)
SSEQ-U + SF-12 (Physical)	*r* = 0.56 (*p* < 0.05)	*r* = 0.49(*p* < 0.05)	*r* = 0.68 (*p* < 0.05)
SSEQ-U + FES	*r* = 0.65 (*p* < 0.05)	*r* = 0.78(*p* < 0.05)	*r* = 0.82 (*p* < 0.05)

[Abbreviations: SSEQ-U = Stroke Self-Efficacy Questionnaire Urdu; FIM = Functional Indpendence Measure; BDI = Beck Depression Inventory; SF-12 = 12-Item Short Form Survey; FES = Fall Efficacy Scale]

Activity domain correlations with FIM (*r* = 0.68), BDI (*r* = 0.34), SF-12 (*r* = 0.5 ), and FES (*r* = 0.65). Self-management domain correlations with FIM (*r* = 0.75), BDI (*r* = 0.27), SF-12 (*r* = 0.49), and FES (*r* = 0.78). Total scores SSEQ-U correlations with FIM (*r* = 0.76), BDI (*r*=-0.54), SF-12 (*r* = 0.68), and FES (*r* = 0.82).

### Internal consistency and test-retest reliability

The internal consistency of each question was > 0.90 which is considered excellent shown in Table 3.

The Test-Retest Reliability of total SSEQ-U was ICC_2,1_=0.956 while in all questions ICC_2,1_ ranged between 0.782 and 0.998. (Shown in Table 3)

**Fig. 2:**
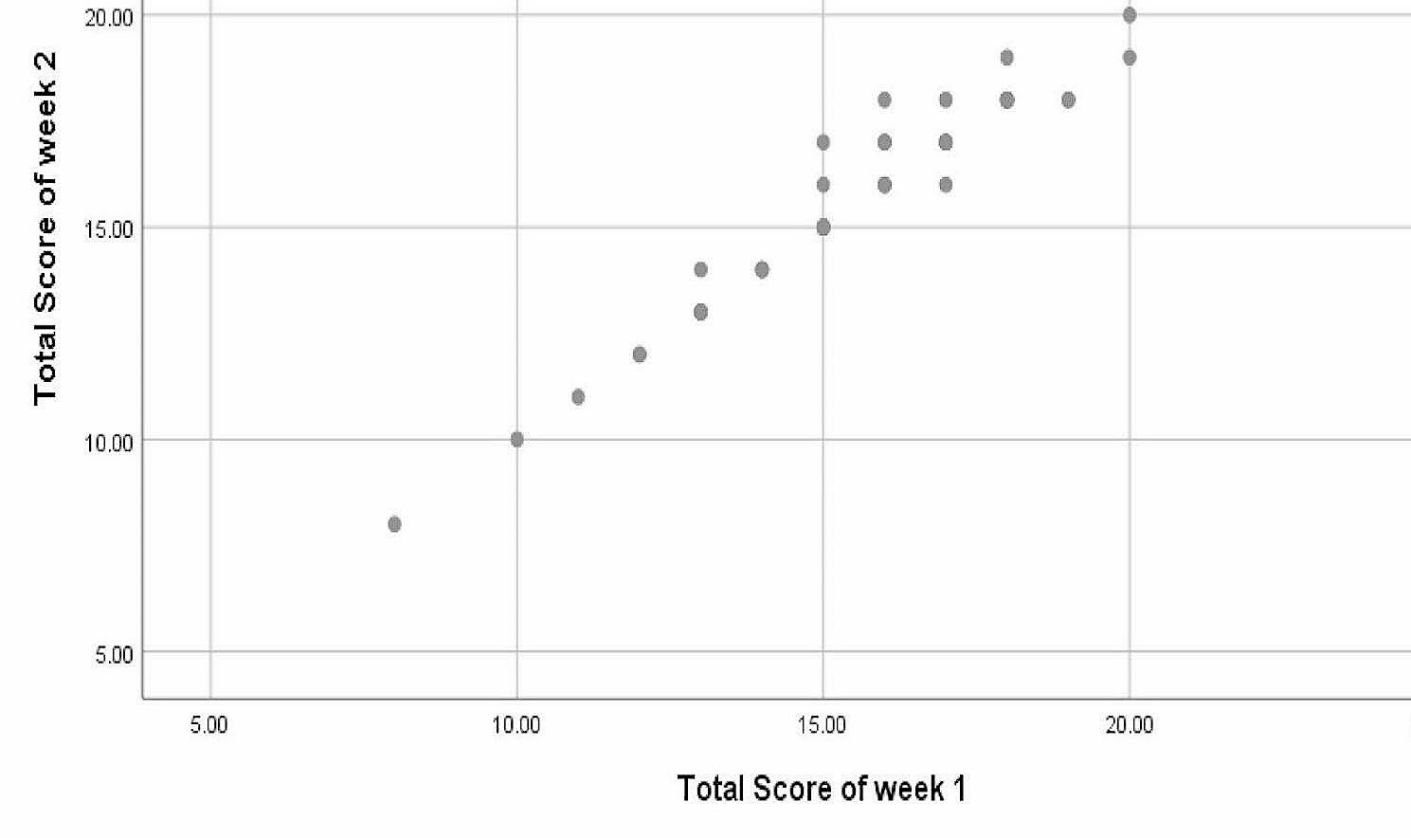
Scatter plot of week 1 and week 2 readings SSEQ-U

Week 1 and week 2 readings of total SSEQ-U were presented as a scatter plot (Shown in Fig. 2).

## Discussion

This study aimed to translate SSEQ into Urdu Language and assess the reliability and validity of the SSEQ-U in stroke survivors. The Stroke Self-Efficacy Questionnaire is intended to measure self-efficacy, a significant influencing factor in stroke survivors’ participation in self-care management and its outcomes.

The pilot study was done to evaluate the translated Urdu statements by the patients, and no major issue was reported. The main purpose was to assess; whether the translated questionnaire was comprehensible and appropriate terminologies for Urdu-speaking nations hence a final Urdu version of SSEQ was drafted. In different versions, a pilot study was done from samples of 10 to 15 participants and also considered face validity. The content validity Index of 13 items was greater than 0.80 which is considered good rated by the six physiotherapists [35–37]. Concurrent validity was determined with other scales. SEM, MDC, ICC and Cronbach’s alpha were calculated to determine the measurement error, test-retest reliability and internal consistency. Measurement error was determined by using two measures; SEM and MDC. SEM is closely associated with error variance, which indicates the amount of variability in a test score administered to a group that is caused by measurement error (level of significance was α < 0.05). MDC is the minimum change in the measure that can be interpreted as a real change (95% Confidence Interval). SEM and MDC for total SSEQ-U in week 1 (0.39, 1.52 points) and in week 2 (0.35, 1.37) respectively that shows minimal acceptable change. In Pourtugese/Brazilian version the SEM and MDC was greater than the Urdu version (SEM was 1.58 points and the MDC was 4.38 points of SSEQ-B).

Concurrent validity was determined by correlating the SSEQ-U to other tools (FIM, BDI, SF-12 and FES). There was an inverse correlation between BDI and SSEQ-U (*r* = − 0.56 *p* < 0.05), suggesting that the more depressive symptoms the individual exhibits the lower the overall self-efficacy while with other scales the correlation was positive. These results are comparable with other versions of the SSEQ (Shown in Table 5). Italian version [20] showed a negative correlation with the Geriatric Depression Scale (GDS) [47], Chinese version [21] showed a moderate correlation with the General Self-Efficacy Scale (GSES) [48] and Portugal and Brazilian versions of SSEQ [24] show positive moderate correlations with FIM (*r*=-0.43), BDI (*r* = 0.52) and Stroke Impact Scale (SIS) (*r* = 0.64) [49].

Comparing the SSEQ-U to other versions in various languages reveals this study’s highest internal consistency and test-retest reliability (Shown in Table 5). The internal consistency of the SSEQ-U (activity domain, self-management and total score) has Cronbach’s alpha > 0.80 which is considered good. Test-retest reliability of total SSEQ-U was ICC = 0.956 and each question has > 0.85. The result seems comparable to all the other versions and with the original version.

**Table 5:** Cronbach’s Alpha and ICC values of different SSEQ versions

Language	Internal Consistency (α)	Test-Retest Reliability (ICC)	Correlations with other scales
English (original) (2008) by Fiona Jones	0.90	0.93/0.91	FES, *r* = 0.803, *p* < 0.001
Chinese (2016) by Suzanne Hoi Shanlo	0.92	0.52	GSES, FAI, SSQOL, *r* = 0.48–0.68,*p* < 0.01
Italian (2016) by Laura DALLOLIO			Activity Factor Correlations= MBI (r-0.46, *p* < 0.001; GDS (*r*= -0.10, *p* = 0.234; Self-Management Factor Correlations= MBI (*r* = 0.21, *p* = 0.009); SF-12-P (*r* = 0.09, *p* = 0.262); GDS (*r* = 0.08, *p* = 0.364)
Denmark (2018) by Lola Quist Kristensen	0.89		
Turkish (2018) Serpil Topcu	0.93	> 0.80	
Portugal-Brazilian (2020) by Marine Portugal Makhoul	0.82, 0.77, 0.68	Intra = 0.91 Inter = 0.81	BDI (*r*= -0.43, *p* = 0.006); FIM (*r* = 0.52, *p* < 0.001); SIS (*r* = 0.64, *p* < 0.001)
Hausa (2021) by Samnu Ali	0.99	0.99	Original (*r* = 0.96, *p* < 0.001)
Brazilian (2022) by Pedro Henrique Deon	0.829		
Arabic (2023) by Fares Almalki	0.93	0.89–0.92	SF-12 (*r* = 0.96, *p* < 0.001)

[Abbreviations: ICC = Intra-class correlation coefficient; FES = Fall Efficacy Scale; GSES = General Self-Efficacy Scale; FAI = Frenchay Acitivity Index; SSQOL = Stroke-Specific Quality of Life; MBI = Modified Barthal Index; GDS = Geriatric Depression Scale; BDI = Beck Depression Inventory; FIM = Functional Independence Measure; SIS = Stroke Impact Scale; SF-12 = 12-Item Short Form Survey]

The Urdu version of SSEQ is as reliable as the Italian version by Dallolio et al. in 2016. There was excellent data quality and acceptability for the SSEQ with good internal consistency [20]. Chinese SSEQ by Suzanne and colleagues (2016) has a very high value of Cronbach’s alpha such as 0.92 and ICC = 0.52 [21]. Internal consistency of the Turkish version of SSEQ by Serdil & Sidika in 2018 shows high internal consistency as Cronbach’s *α* coefficient is calculated as 0.93 and test-retest reliability was found to be high [23]. Portugal-Brazilian version SSEQ in 2020 showed Cronbach’s alpha of 0.82 and test-retest reliability of ICC = 0.81–0.91 [24]. Hausa SSEQ’s internal consistency and test-retest reliability (α & ICC) both were 0.99 greater than the Urdu version and the correlation with the original SSEQ was *r* = 0.96 [25]. The Arabic version of SSEQ results were α = 0.93 and ICC = 0.89–0.92. The correlation with SF-12 was 0.96 greater than the Urdu version of SSEQ [27].

The strength of this study was that it was conducted on a large sample and participants who were in the rehabilitation process were considered in this study. SSEQ-U survey analysis helped stroke survivors to analyze in what domain they had to focus on and in which domain they performed well. These two domains; functional activity and self-management assist clinicians in setting goals on the limitations of the stroke survivors. Using the SSEQ-U is suggested for clinical care purposes and in upcoming research investigations involving stroke survivors. It is recommended to use SSEQ in clinics and research purposes, and it can evaluate stroke self-management intervention effects with more accuracy. SSEQ-U can be used as a primary outcome measure in stroke rehabilitation programs and can monitor physical therapy effects.

There are some limitations of this study, participants with late subacute and chronic stages were involved in this study and this may reduce the generalizability of our findings to acute or early subacute stage. This study did not include anyone with aphasia further limiting the generalizability of our findings to those with speech and language problems. The recruitment strategy that participants already involved in the rehabilitation program was selected for convenience.

## Conclusion

The Urdu version of the Stroke Self-Efficacy Questionnaire was drafted and it was acceptable and accurate for stroke survivors. It showed good content validity, concurrent validity, internal consistency and test-retest reliability.

## Data Availability

No datasets were generated or analysed during the current study.

## Abbreviations

SSEQ: Stroke Self-Efficacy Questionnaire
FIM: Functional Independence Measure
BDI: Beck Depression Inventory
FES: Fall Efficacy Scale
COSMIN: Consensus-based Standards for the selection of health Measurement Instruments
CVI: Content Validity Index
ICC: Intra-class Correlation
